# Trends in early initiation of breastfeeding in Bangladesh and a multilevel analysis approach to find its determinants

**DOI:** 10.1038/s41598-021-84412-5

**Published:** 2021-03-03

**Authors:** Foyez Ahmmed, Muhammad Mahabub Rahaman Manik

**Affiliations:** Department of Statistics, Comilla University, Kotbari, Cumilla 3506 Bangladesh

**Keywords:** Health care, Risk factors, Medical research

## Abstract

Early initiation of breastfeeding (EIBF) is an essential practice for child health as well as for maternal health. This study aims to determine trends, prevalence, and factors associated with EIBF in Bangladesh. Data for this study were extracted from Bangladesh demographic and health surveys (BDHS) 2004, 2007, 2011, and 2014. This study found an increasing trend in EIBF in Bangladesh irrespective of the different characteristics of mothers and children. Chi-square test was conducted to find the association between EIBF and different factors. Multilevel logistic regression analysis was used to consider the hierarchical structure of the data. Regression result showed that educated parents [Adjusted odds ratio (AOR) = 1.14, 95% Confidence Interval (CI) = 1.04, 1.26 ], exposure to media [AOR = 1.13, CI = 1.05, 1.21], 2nd or 3rd birth order [AOR = 1.13, CI = 1.04, 1.23], wanted child [AOR = 1.12, CI = 1.02, 1.23], antenatal visit [AOR = 1.07, CI = 1.00, 1.15], antenatal visit by medically trained provider [AOR = 1.06, CI = 1.00,1.13] and rich wealth index [AOR = 1.10, CI = 1.01, 1.20] were positively associated with EIBF. In contrast, mothers with caesarian delivery [AOR = 0.36, CI = 0.31, 0.40], delivery in private facility [AOR = 0.83, CI = 0.73, 0.95], multiple birth, and higher maternal age were less likely to EIBF.

## Introduction

Breastfeeding provides neonates with all sorts of nutrients like proteins, fats, carbohydrates, and fluids are essential for neonates’ healthy growth and development. American Academy of Pediatrics stated that breastfeeding has immunological, economic, health, nutritional, developmental, psychological, and social benefits^1^. Breastfeeding also has several positive impacts on mothers’ health. It reduces health risks like breast cancer, ovarian cancer, type 2 diabetes mellitus, metabolic syndrome, and cardiovascular disease^2,3^. Early initiation of breastfeeding within one hour after birth provides necessary nutrients with colostrum (first milk) to the neonatal. Colostrum boosts the immune system, growth factor, and other protective factors of the neonates^4^. Studies showed that women who didn’t breastfeed their children within one hour of birth had a higher risk of neonatal death compared to the women who breastfed their children within one hour of birth^5,6^.

Breastfeeding returns the pre-gestation bodyweight of mothers after delivery. Breastfeeding ties a mother and her neonate emotionally. It also reduces mothers’ postpartum depression^7^. Early initiation of breastfeeding reduces postpartum blood loss by releasing oxytocin and helps mothers to produce breast milk. Moreover, breastfeeding has economic importance. A study revealed that investment of USD 1 on breastfeeding returns USD 33^8^. But, only 45% of newborns worldwide and 42% of newborns in South Asia are put to the breast within one hour after birth^9^. In Bangladesh, only 51% of the total newborns were breastfed within one hour after birth^10^. However, Bangladesh has to go a far way to achieve a hundred percent prevalence in EIBF.

Studies conducted on EIBF identified several socio-economic and demographic characteristics of mothers and children associated with EIBF in Bangladesh as well as in other countries. A common practice of those analyses was the use of a single-level logistic regression model. But, the single-level logistic model is not applicable in all cases because of its strict assumptions, specifically, when data contains a hierarchical structure. An alternative approach is the use of multilevel regression analysis. Thus, our study aims to identify trends, prevalence, and factors associated with EIBF in Bangladesh by using multilevel regression analysis in BDHS 2004 to 2014 data since BDHS data contains a hierarchical structure.

## Methodology

### Data collection

Data for this study was obtained from Bangladesh demographic and health surveys (BDHS) 2004, 2007, 2011, and 2014. All of the four surveys were nationally representative cross-sectional surveys, and they collected information on several socio-economic and demographic factors of mothers and children using a two-stage stratified cluster sampling scheme. In the first stage, enumeration areas (EAs) or clusters were selected as primary sampling units (PSU), and in the second stage, households were selected randomly from each of the PSU. However, more details on surveys are available on the publicly accessed surveys reports^10–13^. Information on EIBF in 2004 BDHS were available for all children of mothers born in the past five years of the survey, and information on EIBF in 2007 and 2011 BDHS available for the last child of mothers in the past five years of the surveys. For 2014 BDHS, information on EIBF available for the last child of mothers in the past three years of the survey. However, this study sample included 5214, 4782, 6999, and 4278 women with their last child from 11,440, 10,996, 17,842, and 17,863 women of BDHS 2004, 2007, 2011, and 2014 respectively. Therefore, information of 21,273 women with their last childbirth were used for this study.

### Outcome variable

Early initiation of breastfeeding (EIBF) was the dependent variable for our study defined according to the guideline of WHO^14^. EIBF took value “1” for a child if he/she was put to breasts within one hour of birth, and “0” otherwise. That is, EIBF is a dichotomous variable (1 = early, 0 = delay).

### Covariates

Along with covariates available in different studies, we considered some new covariates for this study. We considered maternal age (≤ 20 years, 21–30 years, ˃ 30 years), place of residence (urban, rural), sex of child (male, female), mode of delivery (vaginal, caesarian), parents’ education (both uneducated, father/mother educated, both educated), Household member (less or equal 5, greater or equal 6), birth order of child (1st, 2nd or 3rd, 4th and higher), Type of birth (single, multiple), mothers age at first birth (less or equal 20 years, greater than 20 years), wanted index child (yes, no), antenatal visit (at least 4, less than 4), antenatal visit by medically trained provider (‘yes’ if mother had at least one antenatal visit by medically trained provider, ‘no’ otherwise), mothers age at first marriage (≤ 18 years, >18 years), delivery facility (public, private, other), and mothers working status (yes, no).

The wealth index of mothers was calculated by principal component analysis based on the resources mother had, and categorized into three categories: poor, middle, rich. Mother body mass index (BMI) was categorized into four categories following the definition of WHO: underweight (< 18.5 kg/m^2^), normal (18.5 kg/m^2^ ≤ BMI < 25 kg/m^2^), overweight (25 kg/m^2^ ≤ BMI < 30 kg/m^2^), and obese (≥ 30 kg/m^2^). Exposure to media defined as ‘yes’ if mother exposed to at least one of the three media: radio, television, and newspaper or magazine; ‘no’ otherwise.

The variable division had six categories for BDHS 2004 and 2007 and seven categories for BDHS 2011 and 2014 as in the 2010 Rajshahi division divided into two divisions: Rajshahi and Rangpur. Hence, to avoid complexity in the calculation, we marge Rangpur division with the Rajshahi division for BDHS 2011 and 2014, and finally, we considered six categories in the variable division: Barishal, Chittagong, Dhaka, Khulna, Rajshahi, and Sylhet.

### Statistical analysis

For trend analysis, prevalences of EIBF were calculated for all the four survey years by different socio-economic and demographic characteristics of mothers and children. Chi-square $\left( {\chi^{2} } \right)$ test was used to identify potential factors associated with EIBF from the pooled data 2004–2014. In order to consider the hierarchical structure in the data, we used a multilevel logistic random intercept model. We considered three models. Model 1 and model 2 were two-level logistic random intercept models. We considered place of residence and division as level-2 factors in model 1 and model 2 respectively. In model 3, we considered a 3-level logistic random intercept model with place of residence as level-2 and division as level-3 factors. For instance, a two-level logistic random intercept model with a fixed level-1 factor has the following formula

1 $${\text{Level}}\,1:\,\,\log \left( {p_{ij} /1 - p_{ij} } \right) = \beta_{0j} + \alpha_{1} x_{1ij}$$

2 $${\text{Level}}\,2:\beta_{0j} = \alpha_{00} + \alpha_{0j}$$

where $p_{ij} = {\text{Pr}}\left( {Y_{ij} = 1} \right)$ with response variable $Y_{ij.}$ However, intra-class correlation (ICC) have to calculate to check the cluster effect and ICC have the following functional formula

3 $$ICC = var\left( {\alpha_{0j} } \right){/}\left( {var\left( {\alpha_{0j} } \right) + \pi^{2} /3} \right)$$

where $var\left({\alpha }_{0j}\right)$ is the variance of random intercept ${\alpha }_{0j}.$ Also, a three-level logistic random intercepts model with a fixed level-1 factor has the following formula

4 $${\text{Level}}\,1:\log \left( {{\text{p}}_{{{\text{ijk}}}} {/}1 - {\text{p}}_{{{\text{ijk}}}} } \right) = { }\beta_{{0{\text{jk}}}} + \alpha_{1} {\text{x}}_{{1{\text{ijk}}}}$$

5 $${\text{Level}}\,2:\,\beta_{0jk} = \beta_{00k} + \alpha_{0j0}$$

6 $${\text{Level}}\,3:\beta_{00k} = \alpha_{000} + \alpha_{00k}$$

where $p_{ijk} = \Pr \left( {Y_{ijk} = 1} \right)$ with response variable $Y_{ijk}$. And, intra-class correlations (ICC) for the above model are calculated as

7 $$ICC_{k} = \frac{{var\left( {\alpha_{00k} } \right)}}{{var\left( {\alpha_{0j0} } \right) + var\left( {\alpha_{00k} } \right) + \left( {\pi^{2} /3} \right)}}$$

8 $$ICC_{J|k} = \frac{{var\left( {\alpha_{0j0} } \right) + var\left( {\alpha_{00k} } \right)}}{{var\left( {\alpha_{0j0} } \right) + var\left( {\alpha_{00k} } \right) + \left( {\pi^{2} /3} \right)}}$$

where $var\left( {\alpha_{0j0} } \right)$ and $var\left( {\alpha_{00k} } \right)$ are the variances of random intercepts $\alpha_{0j0}$ and $\alpha_{00k}$ at level 2 and level 3 respectively. The range of ICC varies 0 to 1. For a given random intercept, ICC value greater than 0 implies the presence of hierarchical structure in the data , hence, should be considered in the analysis^15,16^.

### Ethical approval and consent to participate

Our study used secondary data sets collected by the National Institute of Population Research and Training (NIPORT), Bangladesh, and MEASURE DHS. Ethical approval to conduct the surveys was obtained from NIPORT of the Ministry of Health and Family Welfare, Bangladesh^10–13^. As the study included human participants all legitimate strategies were maintained as of the 1964 Helsinki declaration and its later amendments or comparable ethical standards^10–13^. More detail information are publicly accessible from the website: www.dhsprogram.com. These surveys maintained international ethical standards of anonymity, confidentiality, and informed consent^10–13^. For our study, a latter of data authorization was taken after submitting an online proposal from the Demographic and Health Survey (DHS) Program, ICF International.

### Informed consent

Informed consent has been obtained from all participants associated with the surveys before the commencement of data collection.

## Results

**Figure 1:**
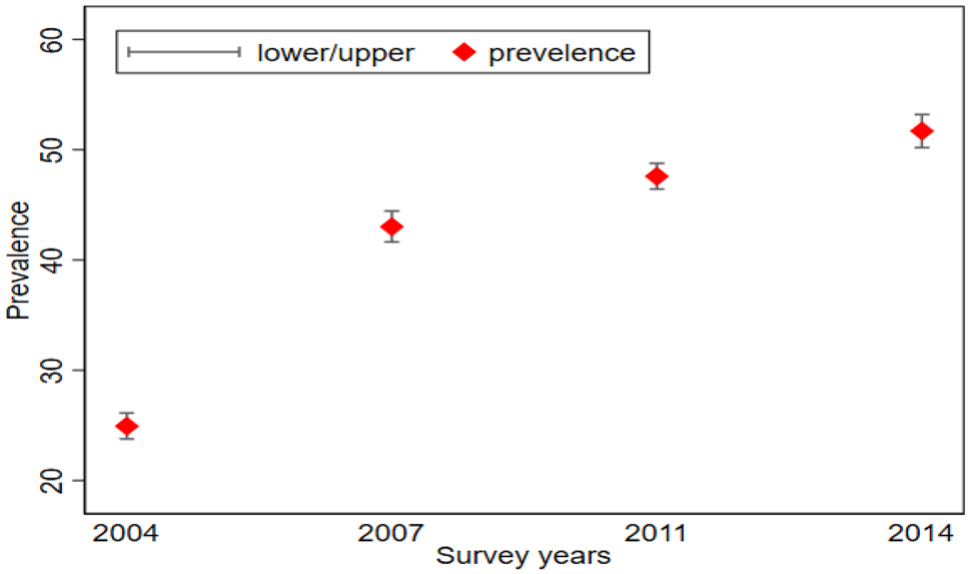
Prevalence of EIBF with confidence interval from BDHS 2004–2014.

We found an increasing trend in the EIBF (Fig. 1) in Bangladesh. Prevalences of EIBF were 24.90, 43, 47.60, and 51.70% in the survey years 2004, 2007, 2011, and 2014 respectively. Prevalences of EIBF by different characteristics of mothers and children were reported in Table 1 for the individual surveys also for the pooled data. Chi-square test was conducted on pooled data to find the association between EIBF and characteristics of mothers and children, and p-values also reported in Table 1. Chi-square test showed that division, place of residence, mode of delivery, parents’ education, number of household members, birth order, type of birth, mothers’ age at first birth, wanted index child, wealth index, delivery facility, and maternal age significantly associated with EIBF. We observed the prevalence of EIBF increased over the surveys for all the categories of covariates related to mothers and their children. For the pooled data, the prevalence was highest in Sylhet (50.50%) and lowest in Chittagong (36.90%). We also found prevalence for the rural area (42.70%) was higher than the urban area (40.10%). Mothers who went under caesarian delivery (28%) had a lower prevalence than the mothers who went under vaginal delivery (44%). Father and mother both educated (43.20%), and either mother or father educated (41.40%) had a higher prevalence than the uneducated parents (37.10%). Prevalence was higher for mothers who lived in households that had less or equal five members compare to their counterparts. Pooled data also showed that 1st birth order (41.90%) and 2nd/3rd birth order (43.40%) had a higher rate of EIBF than the 4th and higher (38.50%) birth order. Mothers who had multiple child-birth had a lower rate of EIBF than mothers who had single child-birth. Surprisingly, we got mothers who had their first birth at less or equal 20 years had a higher prevalence of EIBF than their counterparts. Mothers who wanted the indexed child had a higher rate of EIBF than the mother who didn’t want to. Middle wealth indexed (43.70%) mothers had a higher rate of EIBF than the poor (41.90%) and the rich (40.90%) wealth indexed mother. Mothers who were less or equal to 20 years old (43.90%) had higher prevalence compared to 21–30 years old (42.10%) mothers and more than 30 years old (38.80%) mothers. We also observed that the mothers who had a delivery with a private facility had a low prevalence of EIBF (33%) compared to the mothers who had a delivery with the public (42.80%) and other facilities (43.10%). Mothers who had at least four ANC visits and at least one ANC from a medically trained provider were also found to have higher prevalence of EIBF compared to their opposite counterparts.

**Table 1:** Prevalence of EIBF in different years as well as for pooled data by different covariates, and p-values for the test of association between EIBF and different covariates.

Covariates	Survey years				Pooled	p-value
	2004	2007	2011	2014		
**Survey years**						
2004	24.90					
2007		43.00				< 0.001
2011			47.60			
2014				51.70		
**Division**						
Barishal	22.10	45.40	49.20	53.20	42.70	< 0.001
Chittagong	21.30	34.60	45.20	46.40	36.90	
Dhaka	20.80	42.60	42.90	49.20	37.70	
Khulna	24.60	44.00	41.80	44.40	38.20	
Rajshahi	29.60	43.10	51.20	55.10	45.90	
Sylhet	34.40	51.30	53.50	60.60	50.50	
**Place of residence**						
Urban	24.60	41.70	45.60	47.60	40.10	< 0.001
Rural	25.10	43.80	48.50	53.60	42.70	
**Sex of child**						
Male	25.60	43.70	48.30	50.30	42.10	0.367
Female	24.30	42.30	46.90	53.20	41.50	
**Mode of delivery**						
Vaginal	25.10	44.80	51.40	58.30	44.00	< 0.001
Caesarian	22.10	26.90	27.30	30.80	28.00	
**Parents’ education**						
Both uneducated	20.80	42.80	46.50	55.70	37.10	< 0.001
Father/mother educated	22.70	44.10	48.70	56.10	41.40	
Both educated	27.90	42.70	47.50	50.10	43.20	
**Household member**						
≤ 5	25.50	43.70	48.60	52.30	43.00	0.001
≥ 6	24.40	42.40	46.60	51.10	40.70	
**Exposure to media**						
No	20.90	42.70	47.00	54.50	42.00	0.757
Yes	26.70	43.30	47.90	50.10	41.80	
**Birth order**						
1st	26.70	40.50	46.40	49.70	41.90	< 0.001
2nd or 3rd	26.00	44.30	49.30	52.70	43.40	
4th and higher	21.40	44.00	45.50	54.30	38.50	
**Type of birth**						
Single	25.00	43.10	47.70	51.90	42.00	< 0.001
Multiple	20.80	31.40	31.00	19.00	26.50	
**Mother’s age at first birth**						
≤ 20	24.20	43.90	49.00	52.80	42.30	0.002
> 20	29.10	39.30	41.30	47.10	39.60	
**Wanted index child**						
No	18.10	41.00	46.70	52.50	38.30	< 0.001
Yes	26.20	43.40	47.70	51.60	42.40	
**Antenatal visit**						
Less than 4	23.7	43.90	48.30	53.90	41.80	0.659
At least 4	30.6	40.10	45.60	47.00	42.10	
**ANC by medically trained provider**						
No	21.30	42.80	50.00	55.90	41.50	0.399
Yes	28.30	43.20	45.50	49.50	42.10	
**Body mass index**						
Underweight	24.70	43.30	51.90	54.00	42.10	0.057
Normal	25.00	44.00	46.90	52.60	42.20	
Overweight	26.30	38.10	41.80	46.30	40.10	
Obese	20.40	29.00	45.00	39.10	36.50	
**Mother’s age at first marriage**						
≤ 18	24.40	43.60	48.60	52.60	42.10	0.095
> 18	30.10	39.30	41.90	47.20	40.40	
**Wealth Index**						
Poor	20.30	44.20	48.60	55.20	41.90	0.015
Middle	24.70	43.90	50.90	53.70	43.70	
Rich	29.60	41.50	45.00	47.40	40.90	
**Delivery facility**						
Public	33.30	38.60	45.10	49.20	42.80	< 0.001
Private	26.90	33.30	33.20	34.00	33.00	
Other	24.10	44.50	50.80	58.70	43.10	
**Maternal age**						
≤ 20	27.20	43.80	49.90	53.30	43.90	< 0.001
21–30	24.90	43.80	47.90	50.80	42.10	
> 30	22.70	40.30	44.80	52.30	38.80	
**Mothers’ working status**						
No	25.50	43.10	47.70	51.00	42.00	0.430
Yes	22.40	42.80	46.50	54.40	41.30	

### Multilevel logistic regression analysis

Results obtained from each of the three considered models are reported in Table 2. Besides covariates that had been found significant in the chi-square test, we considered some other covariates in our regression models that had been found significant in other studies. ICC values reported in Table 2 show that we should consider model 3 as our final model as ICC values for level 2 and level 3 random intercepts are greater than zero. We can say EIBF varies among divisions and between the places of residences within a division. We also found that the value of Akaike’s Information Criterion (AIC) slightly greater for model 3 compared to model 2. However, considering both AIC and ICC we considered model 3 as our final model. The regression result showed that the odds of EIBF for all survey years 2007, 2011, and 2014 were greater than the odds of the survey year 2004. Children whose parents (AOR = 1.14, CI = 1.04, 1.26) were educated were more likely to EIBF than the children whose parents were uneducated. Mothers who got caesarian delivery (AOR = 0.36, CI = 0.31, 0.40) had lower Odds of EIBF than the mothers who had a vaginal delivery. In the case of 2nd or 3rd birth (AOR = 1.13, CI = 1.04, 1.23) mothers were more likely to EIBF compared to their 1st birth. We also observed that odds of EIBF was higher for mothers who had at least four antenatal visits during pregnancy (AOR = 1.07, CI = 1.00, 1.15) compared to mother who hadn’t. Mothers with at least one ANC visit from a medically trained provider were found to have higher odds (AOR = 1.06, CI = 1.00, 1.13) compared to their counterparts. Other factors that were significantly positively associated with odds of EIBF include exposure to media (AOR = 1.13, CI = 1.05, 1.21), wanted index child (AOR = 1.11, CI = 1.02, 1.23), and rich wealth index of mothers (AOR = 1.10, CI = 1.01, 1.20). Besides, factors that significantly negatively associated to EIBF included multiple birth (AOR = 0.57, CI = 0.40, 0.82), maternal age [21–30 years (AOR = 0.91, CI = 0.83, 0.99), more than 30 years (AOR = 0.87, CI = 0.76, 0.99)] and delivery in private facility (AOR = 0.83, CI = 0.73, 0.95).

**Table 2:** Adjusted odds ratio for multilevel logistic regression models to EIBF of Bangladeshi children by different socio-economic and demographic factors, BDHS 2004–2014.

Covariates	Model 1^a^		Model 2^b^		Model 3^c^	
	AOR (95% CI)	P_value	AOR (95% CI)	P_value	AOR (95% CI)	P_value
**Year**						
2004^R^	1.00		1.00		1.00	
2007	2.40 (2.21, 2.62)	< 0.001	2.38 (2.18, 2.60)	< 0.001	2.38 (2.18, 2.60)	< 0.001
2011	3.04 (2.82, 3.30)	< 0.001	2.99 (2.75, 3.24)	< 0.001	2.99 (2.75, 3.24)	< 0.001
2014	3.92 (3.57, 4.30)	< 0.001	3.83 (3.49, 4.20)	< 0.001	3.83 (3.48, 4.20)	< 0.001
**Sex of child**						
Male^R^	1.00		1.00		1.00	
Female	0.97 (0.91, 1.02)	0.245	0.97 (0.91, 1.02)	0.266	0.97 (0.91, 1.02)	0.264
**Mode of delivery**						
Vaginal^R^	1.00		1.00		1.00	
Caesarian	0.37 (0.31, 0.41)	< 0.001	0.36 (0.31, 0.40)	< 0.001	0.36 (0.31, 0.40)	< 0.001
**Parents’ education**						
Both uneducated^R^	1.00		1.00		1.00	
Father/mother educated	1.05 (0.95, 1.16)	0.292	1.07 (0.97, 1.18)	0.186	1.07 (0.97, 1.18)	0.182
Both educated	1.10 (1.00, 1.21)	0.042	1.14 (1.04, 1.26)	0.007	1.14 (1.04, 1.26)	0.007
**Household member**						
≤ 5^R^	1.00		1.00		1.00	
≥ 6	0.95 (0.89, 1.01)	0.116	0.95 (0.89, 1.00)	0.060	0.94 (0.89, 1.00)	0.056
**Exposure to media**						
No^R^	1.00		1.00		1.00	
Yes	1.10 (1.03, 1.19)	0.004	1.13 (1.05, 1.21)	0.001	1.13 (1.05, 1.21)	< 0.001
**Birth order**						
1st^R^	1.00		1.00		1.00	
2nd or 3rd	1.14 (1.05, 1.24)	0.001	1.13 (1.04, 1.23)	0.002	1.13 (1.04, 1.23)	0.002
4th and higher	1.13 (1.00, 1.28)	0.044	1.10 (0.98, 1.25)	0.105	1.10 (0.98, 1.25)	0.105
**Type of birth**						
Single	1.00		1.00		1.00	
Multiple	0.57 (0.39, 0.82)	0.002	0.57 (0.39, 0.82)	0.002	0.57 (0.40, 0.82)	0.002
**Mother’s age at first birth (years)**						
≤ 20^R^	1.00		1.00		1.00	
> 20	1.01 (0.91, 1.12)	0.770	1.01 (0.90, 1.14)	0.744	1.00 (0.90, 1.11)	0.916
**Wanted index child**						
No^R^	1.00		1.00		1.00	
Yes	1.13 (1.03, 1.25)	0.007	1.12 (1.02, 1.23)	0.017	1.11 (1.02, 1.23)	0.021
**Antenatal visit**						
Less than 4^R^	1.00		1.00		1.00	
At least 4	1.07 (1.00, 1.15)	0.069	1.06 (0.99, 1.14)	0.115	1.07 (1.00, 1.15)	0.083
**ANC by medically trained provider**						
No^R^	1.00		1.00		1.00	
Yes	1.06 (1.00, 1.14)	0.052	1.06 (1.00, 1.13)	0.072	1.06 (1.00, 1.13 )	0.067
**Mother’s age at first marriage (years)**						
≤ 18^R^	1.00		1.00		1.00	
> 18	1.04 (0.93, 1.17)	0.420	1.01 (0.90, 1.14)	0.744	1.02 (0.91, 1.14)	0.723
**Delivery facility**						
Public^R^	1.00		1.00		1.00	
Private	0.82 (0.71, 0.93)	0.003	0.83 (0.72, 0.94)	0.006	0.83 (0.73, 0.95)	0.006
Other	0.91 (0.81, 1.01)	0.078	0.92 (0.82, 1.01)	0.110	0.91 (0.82, 1.02)	0.100
**Body mass index**						
Underweight^R^	1.00		1.00		1.00	
Normal	0.95 (0.89, 1.01)	0.136	0.97 (0.91, 1.03)	0.384	0.97 (0.91, 1.03)	0.363
Overweight	0.89 (0.80, 1.00)	0.064	0.91 (0.82, 1.02)	0.141	0.91 (0.82, 1.02)	0.144
Obese	0.85 (0.67, 1.08)	0.199	0.87 (0.69, 1.11)	0.304	0.89 (0.70, 1.12)	0.335
**Maternal age (years)**						
≤ 20^R^	1.00		1.00		1.00	
21–30	0.90 (0.82, 0.98)	0.023	0.90 (0.82, 0.98)	0.029	0.90 (0.83, 0.98)	0.029
> 30	0.86 (0.76, 0.98)	0.029	0.86 (0.76, 0.98)	0.031	0.86 (0.76, 0.98)	0.032
**Wealth Index**						
Poor^R^	1.00		1.00		1.00	
Middle	1.06 (0.98, 1.15)	0.124	1.07 (0.99, 1.17)	0.074	1.07 (0.99, 1.17)	0.072
Rich	1.08 (0.99, 1.17)	0.047	1.09 (1.01, 1.17)	0.025	1.10 (1.01, 1.20)	0.018
**Mother’s working status**						
No^R^	1.00		1.00		1.00	
Yes	0.98 (0.91, 1.06)	0.654	0.99 (0.91, 1.06)	0.854	0.99 (0.92, 1,07)	0.881
**Constant**	0.29 (0.23, 0.34)	< 0.001	0.28 (0.22, 0.36)	< 0.001	0.28 (0.22, 0.36)	< 0.001
**AIC**	27,460.55		27,321.75		27,325.44	
**Intra-class correlation coefficient (ICC)**						
Place of residence | division	0.0002465				0.0116557	
Division			0.0105863		0.0102001	
**Random-effect parameters**						
**Estimate (95% CI)**						
Place of residence	.028(.005, .161)				.069(.026, .180)	
Division			.188(.104, .337)		.184(.097, .347)	

^R^Reference category.

^a^Model 1: Two-level logistic regression model considering place of residence as level 2 factor.

^b^Model 2: Two-level logistic regression model considering division as level 2 factor.

^c^Model 3: Three-level logistic regression model considering place of residence as level 2 and division as level 3 factors.

## Discussion

Early initiation of breastfeeding within one hour of birth is one of the effective practices for reducing neonatal mortality and protecting children from some chronic diseases. In Bangladesh, the prevalence of EIBF had increased by almost four folds in 2014 compared to 2004. The prevalence of EIBF almost doubled from 2004 to 2007. This abrupt change might happen because of the increase in the number of higher-educated women. Prevalence of secondary completed or/and higher educated mothers increased from 5.6% in 2004 to 11.9% in 2007^10,11^. Another plausible reason for this sudden increase could be the introduction of the National Nutrition Program (NNP) in 2004 that promote EIBF as an indicator to be achieved. In 2014, the rate of EIBF in Bangladesh (51.7%) was in “good” condition according to WHO^17^. However, the prevalence of EIBF was far-reaching to universal practice.

Three-level logistic regression revealed that parents’ education was one of the key factors for increasing the EIBF rate. We found children with fathers and mothers both educated had a higher chance of EIBF than the children with uneducated parents. This result is very obvious as educated parents have good knowledge of health, therefore, conscious about their children's health. A similar result was found in some other studies^18–20^. We also found that mothers who exposed to media were more likely to initiate breastfeeding within one hour of birth. This may be because of different messages given in the mass media regarding the importance of EIBF motivate mothers to initiate breastfeeding within one hour of birth.

Like some other previous studies, we found caesarian section delivery negatively associated with early initiation of breastfeeding^18,21–24^. The plausible explanation of this finding may be complicacies in caesarian section delivery. As most of the operations are conducted in anesthesia it becomes difficult for mothers to recover from anesthesia within one hour of birth and put their children to the breast. Also, maternal tiredness, respiratory distress among babies, and involvement of obstetricians in different lifesaving activities after surgery are attributable to this finding. Our study revealed a consistent result with some other studies that mothers who got at least four antenatal care visits during pregnancy were more prone to EIBF compared to their counterparts^23,25,26^. This may happen due to the fact that mothers who have ANC visits get instructions of breastfeeding from the physicians, therefore, implicate the instructions. Mothers who received ANC visits from medically trained providers were also found more likely to initiate breastfeeding within one hour of delivery.

We also observed that mothers who lived in rich families had a higher chance of EIBF than mothers who lived in poor families. Mothers who lived in rich families plausibly more educated and had access to mass media, hence, had greater knowledge of breastfeeding compared to mothers who lived in poor families. On the other hand, mothers who lived in poor families probably didn’t have enough money to have health care facilities. This result is consistent with some other studies^21,27^. Mothers who wanted their indexed child were found more likely to initiate breastfeeding within one hour of birth compared to the mothers who didn’t want to. Mothers who didn’t want their index child might not prepare for taking the child, therefore, might not go to physicians. On the other hand, because of the willingness of getting a new baby, mothers might strictly follow the instructions of the health professional for the betterment of newborn health. Multiple births of mothers were found negatively associated with EIBF.

Though babies with second or third birth order had a significantly higher chance of EIBF compared to the babies with first birth order, babies with fourth and higher birth order didn’t show a significant result. It might happen because mothers got experience of child care in their first birth and in the case of fourth and higher birth they plausibly didn’t want the child or might face a lack of resources in terms of time, money, and energy. In consonance, previous breastfeeding experience was found positively associated with intention and initiation of breastfeeding, and the number of children was found positively associated with initiation and inversely associated with intention to breastfeeding^28^. We also found mothers who got delivered in a private facility were less likely to EIBF compared to the mothers who had delivery in a public facility. Caesarian section delivery may be the attributable factor of this finding as we have found from our data about 74% of delivery in the private facility were caesarian section delivery. Another key finding of our study was gender equity in the early initiation of breastfeeding. We found the prevalence of EIBF was slightly higher for males compared to the females for the first three surveys (2004, 200, and 2011), but in BDHS-2014, females exceeded the male. Also, from the regression result, we observed no significant difference in EIBF between males and females. So, we can say Bangladesh met the sustainable development goal 5 (SDG5) – eliminate all forms of discrimination against girls and women, in the case of EIBF.

We found younger women and adolescents girls were more likely to early initiation of breastfeeding within one hour of birth. A similar result was also found in a study of Namibia^23^. The plausible explanation for this finding may be the improvement in girls’ education, the number of planned pregnancies, and the increasing trend in antenatal visits^10–13^.

For some variables, we found results obtained in bivariate analysis reversed in the multivariate analysis. This reversal paradox happens because in the bivariate analysis we only check the association of a covariate with our response variable, but in the multivariate analysis, we find the association of a covariate with our response variable adjusting the influence of other factors^29,30^. Differences in the prevalence of EIBF were observed in different divisions of Bangladesh, and these differences might happen because of the availability of NNP in different Thanas (sub-district). But we weren't able to evaluate that as in our data there was no information about whether NNP was available for a mother or not. Difference in EIBF was also found in places of residence within divisions. However, differences in the prevalence of EIBF in different geographical regions and places of residence might happen because of the difference in the health facilities, access to healthcare-related information, and access to media^10–13^.

## Conclusion

It is well established that EIBF reduces child mortality and morbidity to a certain extent. It is optimistic that EIBF was in an increasing trend over the last years. However, the prevalence of EIBF is still far-reaching to a "very good" (90 to 100%) state^17^. Achieving a very good state in EIBF will help to achieve sustainable development goal 3 (SDG3) in Bangladesh since one aim of SDG3 is to reduce neonatal mortality to below 12 per 1000 live births and under-5 mortality to below 25 per 1000 live births. Our study revealed all factors that positively associated with EIBF and negatively associated as well. This study calls for the greater attention of concerned authorities in Bangladesh to come up with appropriate policy interventions to increase the prevalence of EIBF and reduce associated risks to it.

## Data Availability

The study conducted based on data collected from Bangladesh Demographic and Health Survey (BDHS). Information on data can be accessed at https://dhsprogram.com/data/available-datasets.cfm.
